# 

**Published:** 1916-01

**Authors:** 


### CONTENTS.

ORIGINAL COMMUNICATIONS—

Presidential Address to Fulton County Medical Society. Taking Stock, by W. A. Selman, M. D. ... 467

A Resume of the Present Status of the Cancer Problem, by Marion McH. Hull, M. D., Atlanta, Ga. ... 470

Diarsenol in the Treatment of Syphilis: Clinical Report, by E. P. Merritt, M. D., Atlanta, Ga. ... 475

Letter From a Subscriber ... 478

EDITORIAL ... 482

SELECTIONS AND ABSTRACTS ... 491

BOOW REVIEWS ... 510

